# Analysis of electric scooter user kinematics after a crash against SUV

**DOI:** 10.1371/journal.pone.0262682

**Published:** 2022-01-21

**Authors:** Mariusz Ptak, Fábio A. O. Fernandes, Mateusz Dymek, Christopher Welter, Kacper Brodziński, Leszek Chybowski

**Affiliations:** 1 Faculty of Mechanical Engineering, Wroclaw University of Science and Technology, Wrocław, Poland; 2 TEMA—Centre for Mechanical Technology and Automation, Department of Mechanical Engineering, University of Aveiro, Aveiro, Portugal; 3 Aachen Institute for Advanced Study in Computational Engineering Science, University: RWTH Aachen, Aachen, Germany; 4 Faculty of Marine Engineering, Maritime University of Szczecin, Szczecin, Poland; Tsinghua University, CHINA

## Abstract

The article presents the results of the analysis of electric scooter user kinematics after a crash against a vehicle. The share of electric scooters (e-scooters) in urban traffic has been growing in recent years. The number of road accidents involving e-scooters is also increasing. However, the safety situation of electric scooter users is insufficiently researched in terms of kinematics and injury outcomes. The article presents the importance of this problem based on an in-depth literature analysis of e-scooter-related types of accidents, injuries percentages, and helmet use. Subsequently, four accident scenarios were designed and simulated using two numerical codes–LS-DYNA for handling finite element (FE) code (the vehicle and scooter model) and MADYMO for multibody code (dummy model). Scenario one is a side bonnet crash that simulates an accident when the scooter drives into the side-front of the vehicle. The second and the third simulation is a side B-pillar crash, which was divided into two dummy’s positions: the squat and up-right. The fourth simulation is a frontal impact. For each scenario, subsequent frames describing the dummy movement are presented. The after-impact kinematics for various scenarios were analyzed and discussed. The plots of the dummy’s head linear acceleration and its magnitude for the analyzed scenarios were provided. As the study is devoted to increasing riders safety in this means of transportation, the potential directions for further research were indicated.

## Introduction

Traffic congestion is a worldwide burden, with issues ranging from stressful driving to increased CO_2_ emissions and a decrease in air quality. Micromobility is growing rapidly in urban road networks, from human-powered to electric vehicles. The usage of electric-powered vehicles, such as standing scooters, is increasing at a rapid pace, especially after the introduction of shared e-scooter services. In 2018 alone, 38.5 million trips using a standing electric scooter (SES) sharing service were reported [1]. This form of transportation seems to have been a practical solution for short-distance commuters, as it is not only sustainable but also a convenient form of mobility.

**Fig 1:**
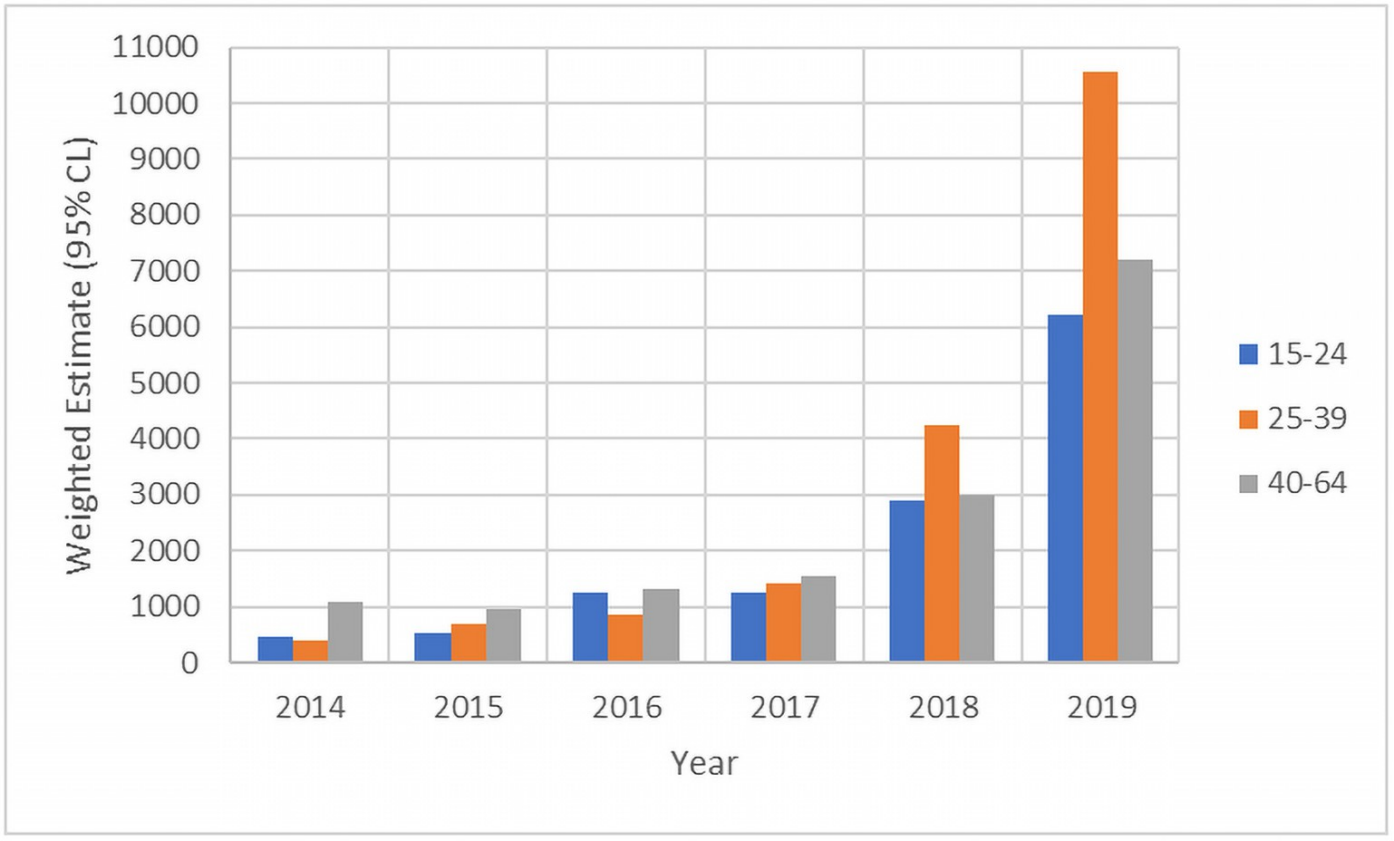
Trends in weighted incidence of electric scooter-related injuries seen in emergency departments in the United States from 2014 to 2019 by selected age groups [4].

Although e-micromobility has a positive impact through decreasing traffic congestion and hazardous emissions, there are disadvantages. The downside of the increase of portable e-vehicles is the increase of injuries and fatalities among users, as shown in Fig 1. Considering SES, Trivedi et al. [2] reported head injuries as the most common injury, followed by fractures and skin abrasions and lacerations. It was also reported that less than 5% of SES riders wore a helmet [1] from 249 injured patients involved in SES accidents between 1 September 2017 and 31 August 2018 in California. In another study in Auckland, the introduction of shared SES is also correlated with a large number of serious related injuries [3], reporting 64 patients between 15 August 2018 and 15 December 2018. From the 64 injuries, the severity is clear: 27 limb fractures, 3 dislocations, a fractured spine, 12 patients with concussion, 1 extradural bleed and 9 facial or skull fractures [3]. Additionally, multiple soft tissue injuries were reported, with 40% of the patients requiring admission to a speciality service and imaging, and 25.4% requiring operative intervention [3].

In another study from Denmark where 468 scooter-related injuries were recorded, Blomberg et al. [5] concluded that current usage rules might not prevent unnecessary accidents and secure traffic safety and the lives of older individuals. The use of a helmet was also discussed by Blomberg et al. [5] as in other previous studies [2, 3, 6]. Blomberg et al. [5] indicate that an entirely different pattern of injuries is emerging. Adults riding faster scooters are resulting in high energy impacts with 20.5% of riders sustaining head injuries. As a result, Blomberg et al. [5] recommend helmet usage.

Similar conclusions were drawn by Kobayashi et al. [7], reporting the increase of trauma cases related to SES accidents. Over half of the patients (51% of 103 patients) presented significant injuries, including intracranial haemorrhage and fractures requiring surgery. Additionally, Kobayashi et al. [7] reported the common use of alcohol and illicit substances and the infrequent helmet use, highlighting the need to increase helmet use and discouraging intoxicated driving among electric scooter users. Table 1 presents a brief review of four studies in the literature that reported the number of injured riders, average age, the number of facial and head injuries, the number of traumatic brain injuries (TBI), the number of riders wearing a helmet and the cause of the accident.

**Table 1:** A summary on e-scooter-related types of accidents, injuries percentages and helmet use, NR—Not reported.

Reference	Blomberg et al. [5]	Störmann et al. [8]	Farley et al. [4]	Austin Public Health [9]
Data collection period	2016–2019	2019	2014–2019	Sep-Nov 2019
Number of injured	112	76	70644	190
Average Age (years old)	27	28–34	29–33	27
Share of accidents with head injury [%]	20.50	17.10	27.10	48.00
Major head injury (resulting in TBI) [%]	11.25	11.50	13.55	21.50
Share of accidents where a helmet was worn [%]	3.60	1.30	1.70	NR
Share of accidents associated with moving object [%]	8.90	8.00	NR	NR
Share of accidents associated with facial injuries [%]	38.40	21.10	NR	NR

Recently, Toofany et al. [10] reviewed the current literature addressing trauma outcome from road accidents involving SES. It was highlighted that the head, upper extremities and lower extremities are particularly vulnerable in SES falls or collisions, while injuries to the chest and abdomen are less common. In the same study, Toofany et al. [10] highlighted the low rates of helmet use among SES users.

Motor vehicles are involved in about 80% of crashes resulting in the death of bicycle or SES riders, and over 80% of these deaths result from crashes with heavier vehicles [11]. Trivedi et al. [2] reported falls and collisions as the most common mechanism of injury involving SES drivers. In the literature, to the best of our knowledge, there is no study reporting accident reconstruction or simulation of accidents involving SES. This is in direct contrast to other vulnerable road users (VRUs) [12, 13]. This is a surprise since recent studies report an alarming number of injuries among scooter drivers, including traumatic brain injuries. Nevertheless, Xu et al. [14] assessed the head injury risk of four types of VRUs during vehicle crashes; pedestrians, bicycles and self-balancing electric scooters (solo-wheel and double-wheel). This was accomplished using MADYMO software, which has been a prevalent numerical simulation program used to study crash safety during accidents in previous works. Xu et al. [14] motivation was based on the fact that electric self-balancing scooters were widely used by commuters. It was necessary to study the safety of this means of transport as previously for other VRUs [12, 13, 15–17].

**Fig 2:**
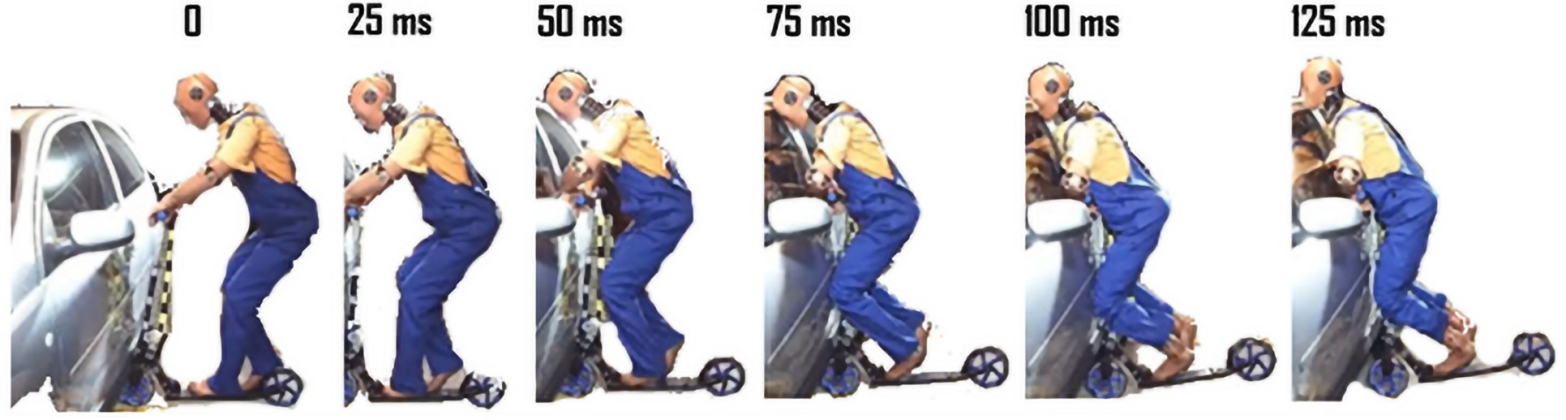
Scooter-to-B-pillar impact—Physical experiment depicted in 25 ms intervals; authors’ illustration based on published fka company materials [18].

The scooter accident scenarios were already investigated by some research groups [10, 18]. The fka company with the Institute for Automotive Engineering (ika) of RWTH Aachen University presented a test series with possible collision scenarios of vehicles and scooters. In Fig 2, there is a 150 ms (with 25 ms intervals) side crash test presented with the SES impact velocity of 24.8 km/h.

Although a few attempts have been made to reconstruct road accidents involving SES, as illustrated in Fig 2, there is the need for deeper investigations and studies of the potential injurious scenarios involving SES drivers. In the literature, numerical models are usually used to study road accidents involving micro-vehicles (vehicles under the micromobility scope such as bicycles). However, to the authors’ best knowledge, this is the first reported model of an SES and also to employ it in the simulation of road accidents. This research makes it possible to identify the risk of several accident configurations, which can give insights on the risks of driving positions, impact speeds, impacted surfaces/structures, and other variables of a road accident involving an SES user.

Therefore, this study aims to evaluate the safety of SES users. The goal of the present study is to simulate road accidents involving SES, determining plausible impact scenarios and thus generating data to develop guidelines for future regulation and safety gear. First, a numerical model of an SES was developed by reverse engineering. Then, this model was used to simulate four different car accident scenarios: frontal impact, side bonnet impact, and impact against B-pillar in two different user positions.

## Materials and methods

### Data of analyzed scooter and numerical approach

In the study, we use a numerical model of a popular Lime company electric scooter obtained through the reverse engineering method [19, 20]. The 3D geometric model was developed with the aid of a Leica P20 laser scanner. The scanned SES was then exported to stereolithography format (STL), which enabled us to proceed with digital processing using computer-aided design (CAD). The geometry was divided into 3 parts of differing thicknesses named: main tube, plates and remaining. The material characteristic was verified using a handheld X-ray Bruker spectrometer. The scooter was checked in 4 different areas to obtain manufacturing materials. The results of the spectroscopy investigation showed an aluminium alloy (Aluminum 6061) as the primary body material. The mass is based on the weights of multiple scooters and is evaluated at 14 kg. The thickness of the parts was measured by the ultrasonic thickness measuring gauge.

The vehicle used in this study was Toyota RAV4 finite element model developed by the FHWA/NHTSA National Crash Analysis Center at George Washington University. The model was successfully validated [21].

**Fig 3:**
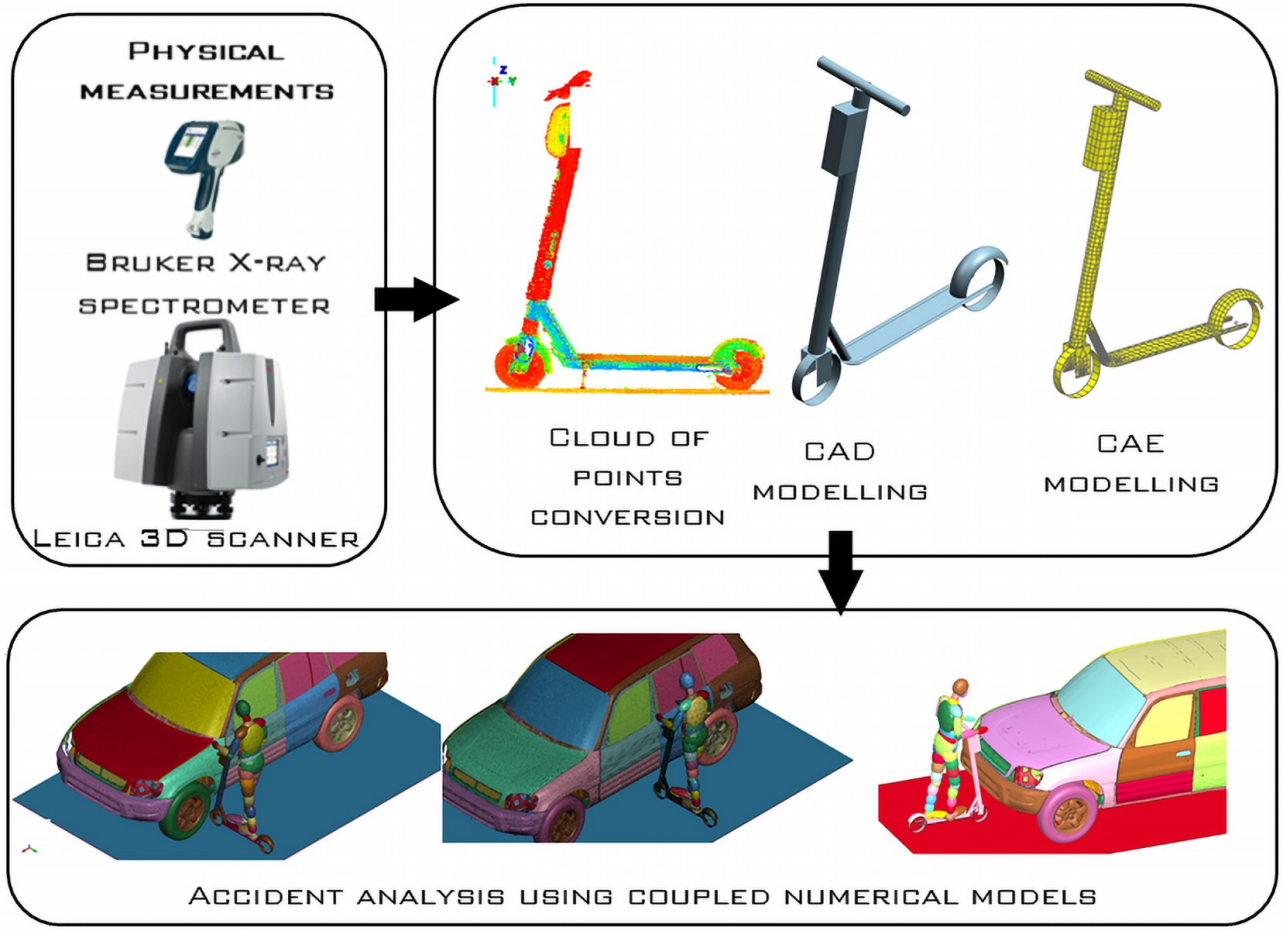
The approach used for the study was based on reverse engineering and coupled LS-DYNA with MADYMO codes.

The full model setup encompasses two numerical codes–LS-DYNA for handling finite element (FE) code (the vehicle and scooter model) and MADYMO for multibody code (dummy model)—Fig 3. The combination of both codes via an interface during the simulation enabled the author to have recourse on benefits, mainly accuracy and biofidelity of a human model, which are not available in a stand-alone approach [22, 23]. The code linkage of LS-DYNA with MADYMO called coupling is a robust practice to solve simulative problems in the area of crash analyses, particularly under consideration of involved vulnerable road users. The Hybrid III-dummy in the 50^th^ percentile male version is the commonly implemented MADYMO dummy in the area of automotive safety device evaluation [24, 25], and it was also used for the presented set of simulations. The dummy model is the ellipsoid pedestrian model developed by TNO Automotive. The model is one of the most frequently used multi-body pedestrian models for vulnerable road user crash reconstruction [26–28]. The interaction between MB–instances and–parts of the scooter and the dummy versus the FE–instances and parts of the vehicle, were installed by a *coupling* and the sub-elements of *contacts* and *restraints* in both MADYMO and LS-DYNA codes. The dummy is validated for each model segment such as tibia, femur, pelvis, thorax, shoulder and for the full model [29, 30]. The disadvantage is that the validation is only for vehicle impact and not for ground contact [31–34]. Nevertheless, this study is focused only on pedestrian-vehicle contact and not on pedestrian-ground contact after the impact. Therefore, it is a suitable and valid model to be employed in this study.

The model of material used in LS-DYNA for the numerical model of the scooter is depicted in Table 2. The numerical components, their physical properties and applied numerical approach for the study are presented in Table 3.

**Table 2:** The material model for aluminum 6061 used for the scooter.

Density [kg/m^3^]	Young’s Modulus [GPa]	Poisson’s Ratio	Yield Stress [MPa]	Tangent Modulus [MPa]
2700	69.8	0.33	178.7	691

**Table 3:** The numerical components, their physical properties and applied numerical approach for the study.

Numerical Components		Physical Properties	Numerical Approach
Vehicle		Mass: 1266 kg Wheelbase: 2415 mm	Toyota RAV4 model developed by the FHWA/NHTSA National Crash Analysis Center at The George Washington University [21]
Dummy		Mass: 75.7 kg Height: 1.74 m	MADYMO multibody 50th percentile male model developed by TNO Automotive
Scooter	Main Tube	Thickness: 5 mm Aluminium 6061	#24530 shell first-order finite elements–a piecewise linear plastic model of material
	Deck	Thickness: 4.5 mm Aluminium 6061	#9544 shell first-order finite elements–a piecewise linear plastic model of material
	Other parts	Thickness: 2 mm Aluminium 6061	#36545 shell first-order finite elements–a piecewise linear plastic model of material
	Wheels	Rigid tire	Rigid bar element 2 (RBE2) from the beam with released rotational degrees of freedom (wheel axis) to the rigid tire shell elements
	Battery	Mass: 2 kg	0D mass element added to the centre of gravity (CoG) of the battery and connected by RBE2 to the main tube

### Description of scenarios

**Fig 4:**
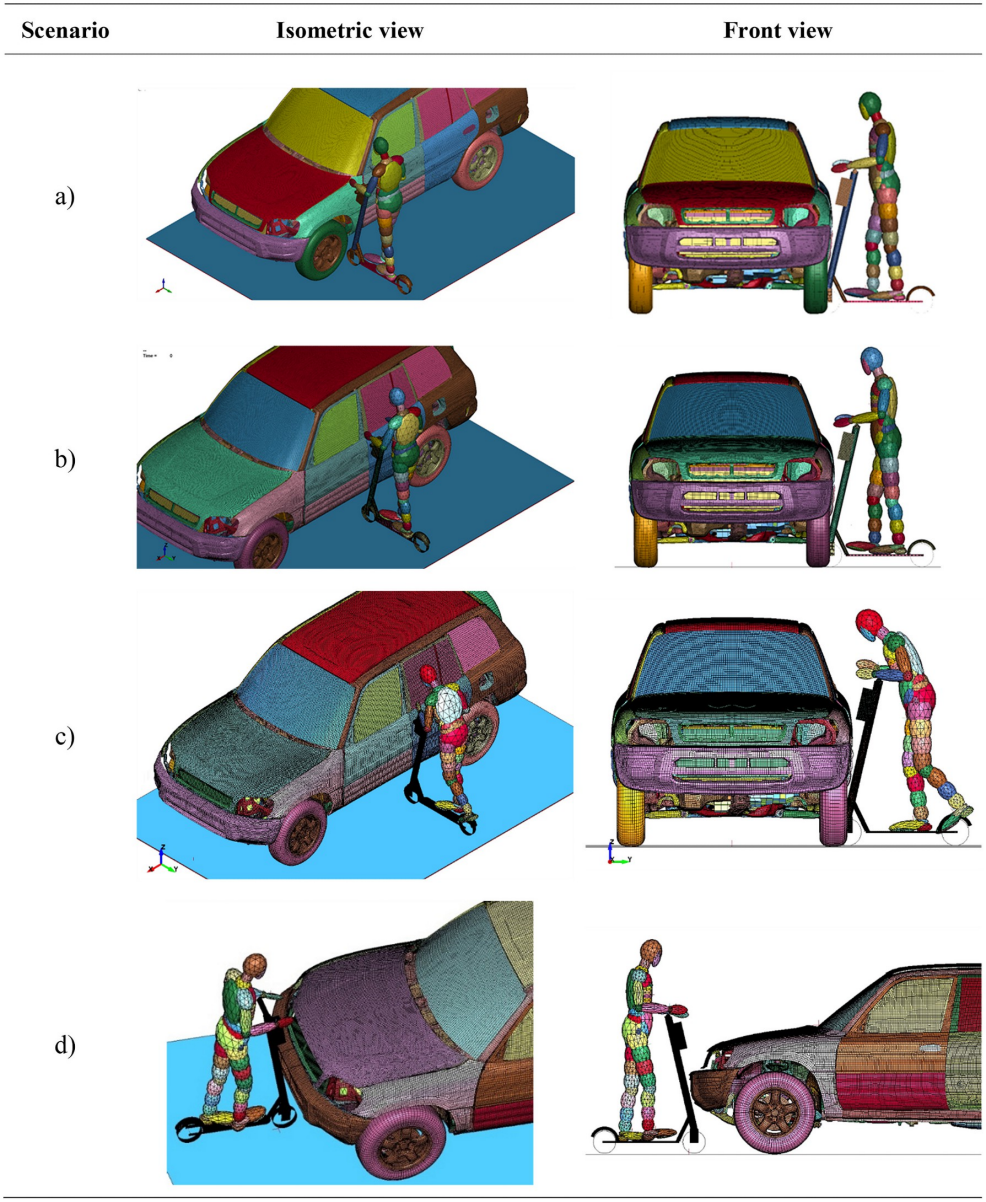
Impact scenarios: a) Bonnet impact b) side impact B-pillar upright position c) side B-pillar squat position d) symmetrical frontal impact.

Electric scooters, despite their many advantages, such as ease of movement, ecology and economy, also have many disadvantages. One of them is safety issues. In theory, scooters are designed to be durable and safe, although a human plays a significant role in safety. There were four different simulation setups analyzed in this study (Fig 4). The first analysis (scenario a)) is a side bonnet crash that simulates an accident when the scooter drives into the side-front of the vehicle. This case is expected to have the most unpredictable kinematics.

The second and the third simulation is a side B-pillar crash as the B-pillar is one of the stiffest parts on the side of a vehicle. Such an accident may carry enormous consequences. The B-pillar crash was further divided into two dummy positions: up-right (scenario b)) and squat (scenario c)). Squat means that the dummy’s knees are slightly bent, whereas up-right means that the leg is fully extended. This was investigated since the user height has a significant influence on the kinematics—it may be the determining factor deciding whether the rider hits the B-pillar or the hood of the car. The squat position mimics the case presented in the experimental studies depicted in Fig 2.

The fourth simulation (scenario d)) is a frontal impact. This case is expected to be the most dangerous in terms of scooter driver injuries due to the high velocities and thus potential decelerations.

For the B-pillar and side bonnet case, the velocity of the scooter is 25 km/h, whereas, for the frontal impact, the vehicle has a velocity of 21.6 km/h. The vehicle was stationary for all scenarios with tire-ground contact and inflated tires. The assumed crash velocity of 25 km/h for side impacts reflects the maximum allowed cruise velocity for electric scooters in most European countries. Unlike the side-impacts, when the vehicle may drive from a crossroad or an alley, we assumed the braking action of the scooter’s user for the frontal impact–thus, the impact velocity was reduced to 21.6 km/h (6 m/s).

According to literature research and urban accident review, collisions with vehicles, along with falls, are the two major types of accidents involving e-scooters. The presented scenarios are an attempt to represent some of the most likely to occur involving an automobile since e-scooter collisions with vehicles are reported to be mainly on arterial roads/streets and intersections [35]. Of all e-scooter crashes reported in [36], 54% occurred at an intersection with a motor vehicle traveling straight or turning right and an e-scooter rider entering the crosswalk from the right. Moreover, both b) and c) scenarios are based on the crash test presented by the fka company with the Institute for Automotive Engineering (ika) of RWTH Aachen University–depicted in Fig 2.

## Results and discussion

**Fig 5:**
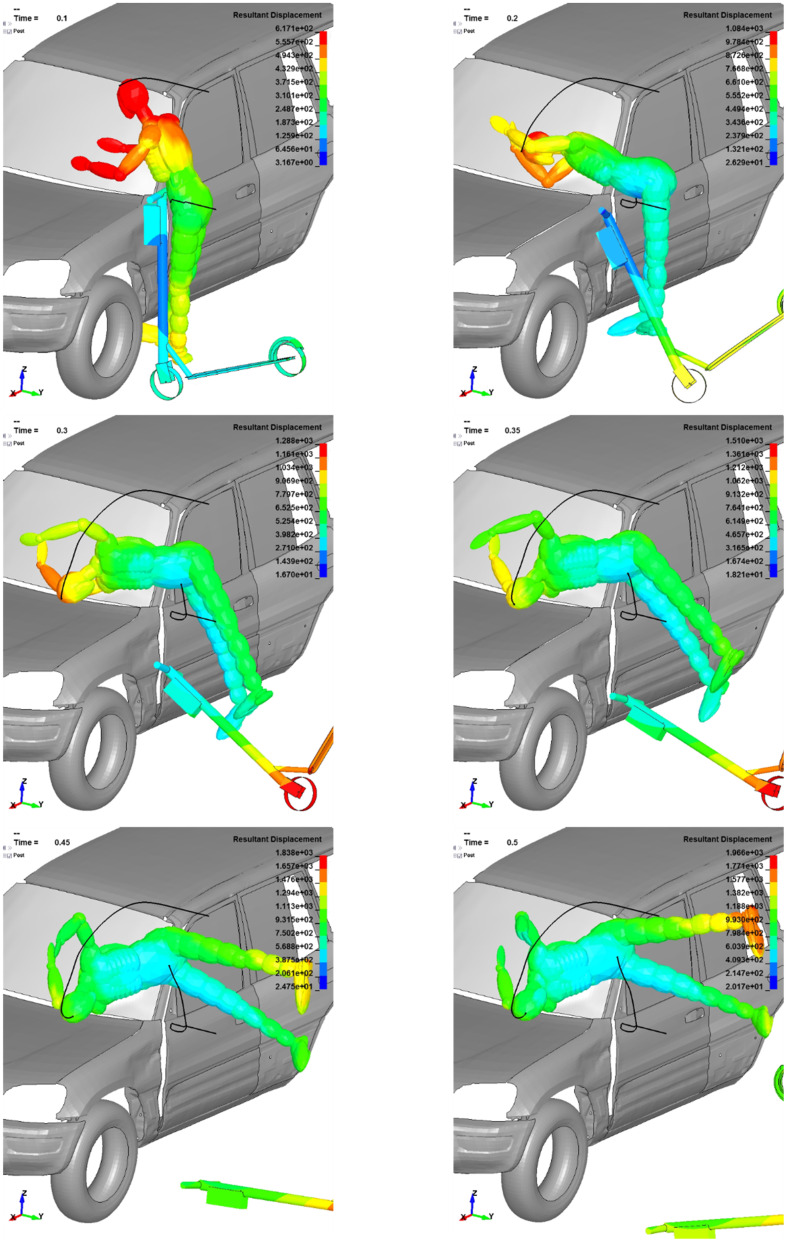
Dummy kinematics in the side bonnet scenario—The dummy’s resultant displacement in [mm], time in [s].

**Fig 6:**
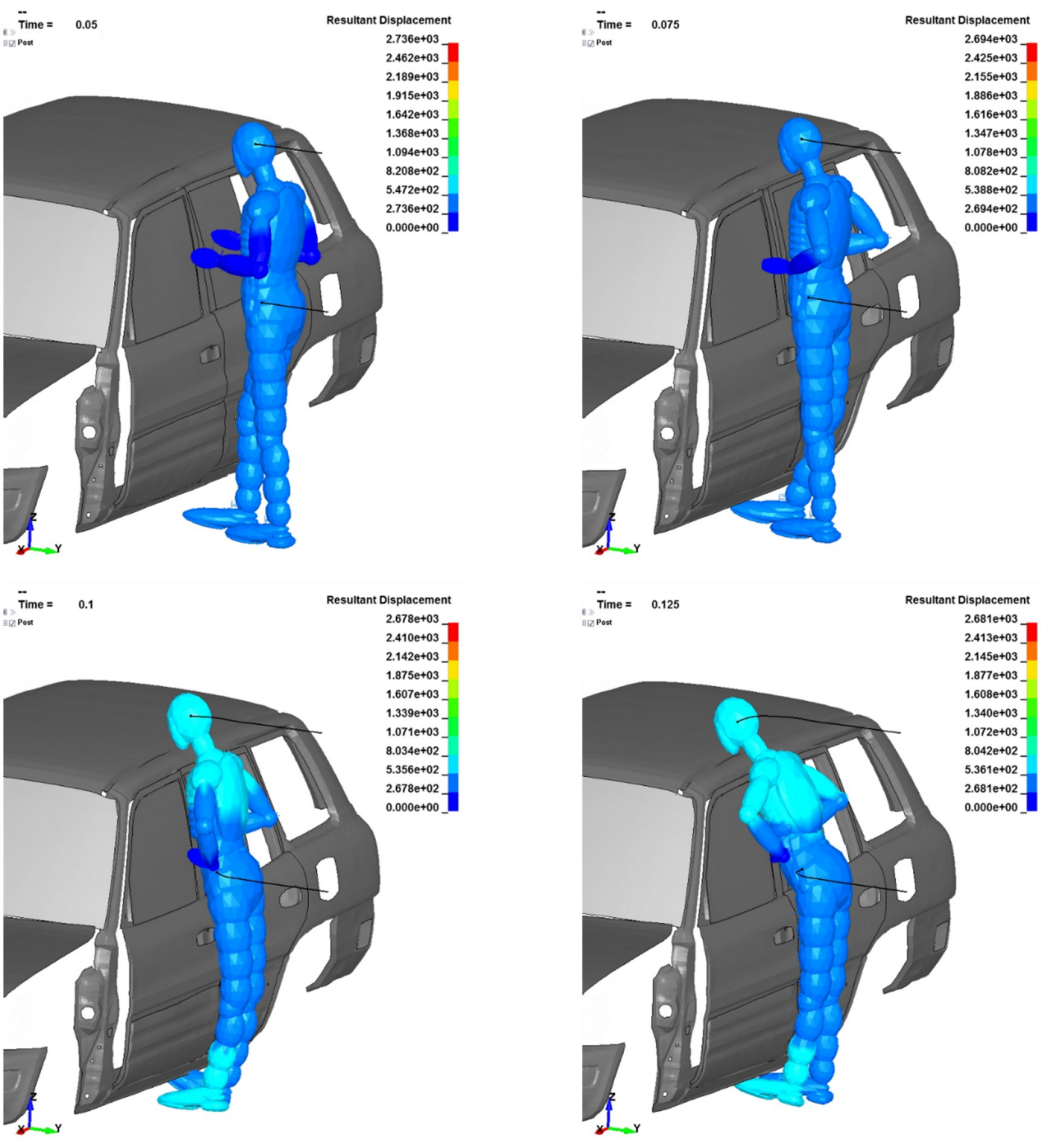
Dummy kinematics in the B-pillar, up-right scenario—The dummy’s resultant displacement in [mm], time in [s].

**Fig 7:**
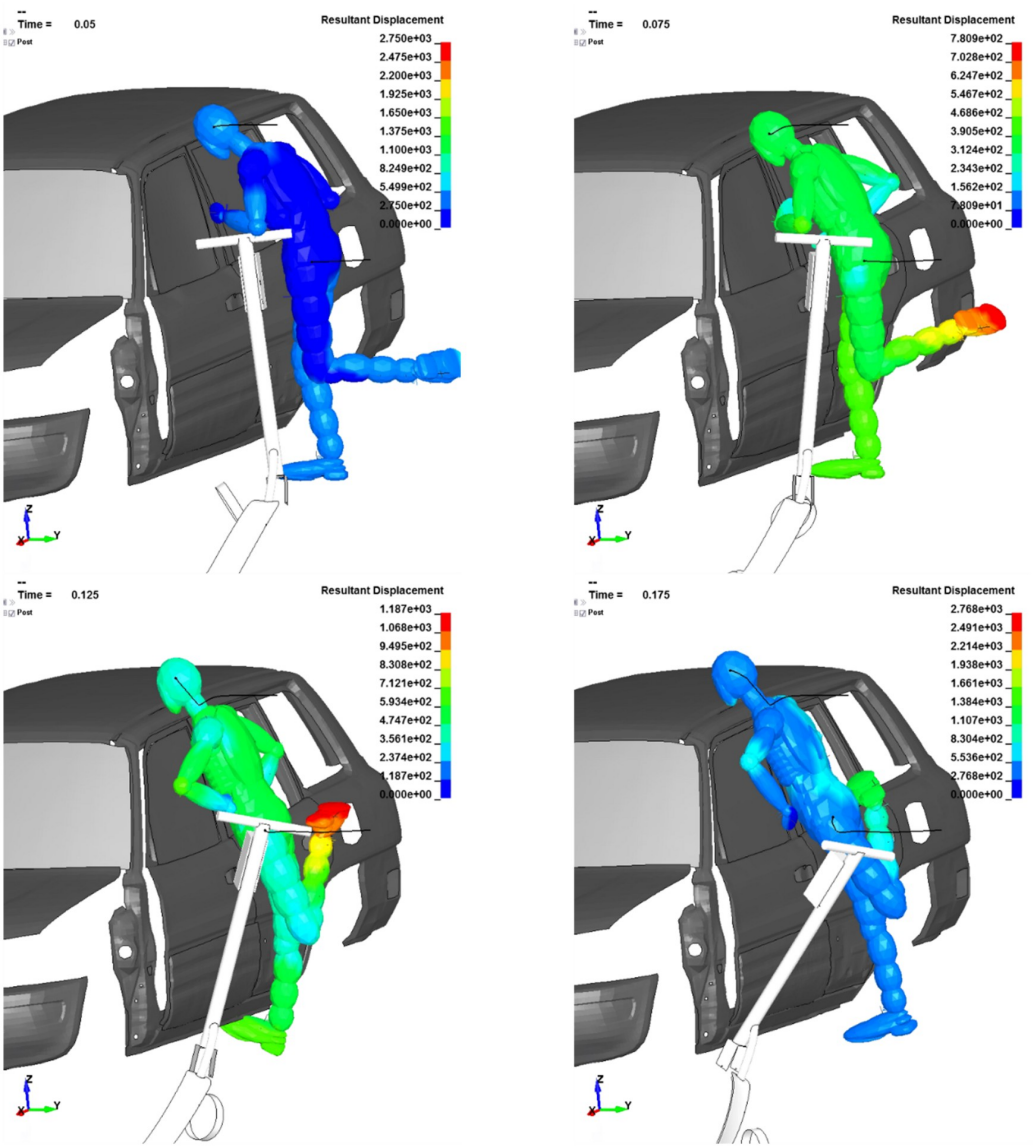
Dummy kinematics in the B-pillar, squat scenario—The dummy’s resultant displacement in [mm], time in [s].

**Fig 8:**
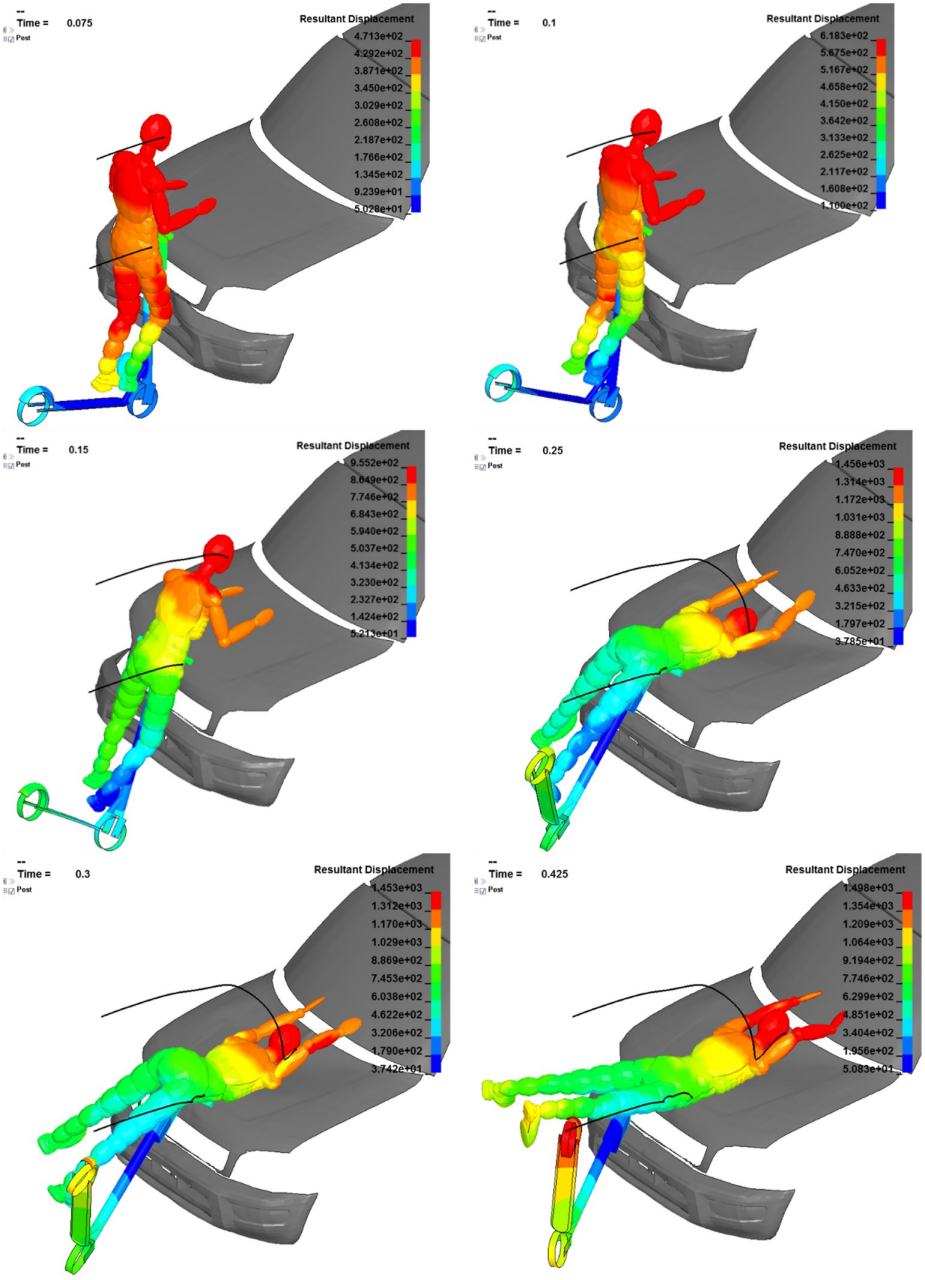
Dummy kinematics in the frontal scenario—The dummy’s resultant displacement in [mm], time in [s].

In the presented scenarios, we focused on the kinematics of the scooter‘s user (the rider) and we tracked the head’s and dummy’s (marked at the time 0, i.e. user-vehicle contact) centre of gravity (CoG). The tracked CoGs of the head and dummy (in the initial position) are depicted as the black curves in the following Figs 5–8. The relative displacement of the user is in [mm], and it relates to time 0 of a simulation.

### Scenario a)—Side bonnet impact

In the side bonnet scenario, the arms behaviour is crucial to investigate. The first user-vehicle contact is at the height of the dummy’s CoG. Further, the right forearm and elbow impact the windshield and protects the head from severe injuries. The dummy arm contact decreases the force impulse and thus decelerating the head. Similar to the subsequent cases, the contact point between the scooter and vehicle acts as the point of rotation, causing a rotation of the dummy. The electric scooter behaves as a rigid body component and does not significantly influence the user’s kinematics after the impact.

### Scenario b)—B-pillar impact, up-right dummy position

In the B-pillar numerical simulation–the up-right position, the major contact is between the B-pillar and the dummy’s torso with legs. As the torso and legs have nearly null velocity components–i.e. the velocity is equalized with the stationary vehicle–the head-neck system is the main part with relatively high kinetic energy and thus suddenly rotates towards the vehicle. The 50^th^ percentile dummy is taller than the vehicle, which leads to the situation when the head is in contact with the roof–there is no direct contact between the head and B-pillar. This has an essential effect on the deceleration of the head as the roof of the car is not as stiff as the B-pillar.

### Scenario c)—B-pillar impact, squat dummy position

In scenario c) the scooter user is in the squat position and the head kinematics is radically different than in the up-right position presented above (scenario b). The main impact is between the head and the top of the B-pillar. This leads to an opposing situation as in case b), as now there is no constant point of rotation for the head on the car. After the initial contact, the head begins to slide towards the roof–this behaviour is also seen in the FKA experimental crash-test (Fig 2). The leg-torso behaviour is similar to the up-right position–it impacts the vehicle’s side doors and the lower body stops momently. Nevertheless, in both dummy setups, the probability of head injury is very high as there are minimal chances for the user to use their arms to protect the head from direct cranial impact.

### Scenario d)—Frontal impact

The contact in the frontal setup is initiated by the scooter to the front-end of the vehicle. The user maintains the kinetic energy and is forward projected. With the contact to the steering rod, the scooter is pushed by the user and falls onto the bonnet. This contact point between the scooter and the vehicle acts as the dummy’s temporary axis of rotation. The dummy thus rotates around this axis until it hits the middle of the bonnet. During the simulation, it is visible that the first contact between the dummy and bonnet is initiated by the arms. When analyzing the acceleration of the head’s CoG, it shows that this contact decreases the kinetic energy, thus leading to less head inertia if and when the head contacts the bonnet directly. In fact, this situation can closely mimic reality as the user will likely protect their head with an involuntary reflex.

### Comparative analysis of the scenarios

**Fig 9:**
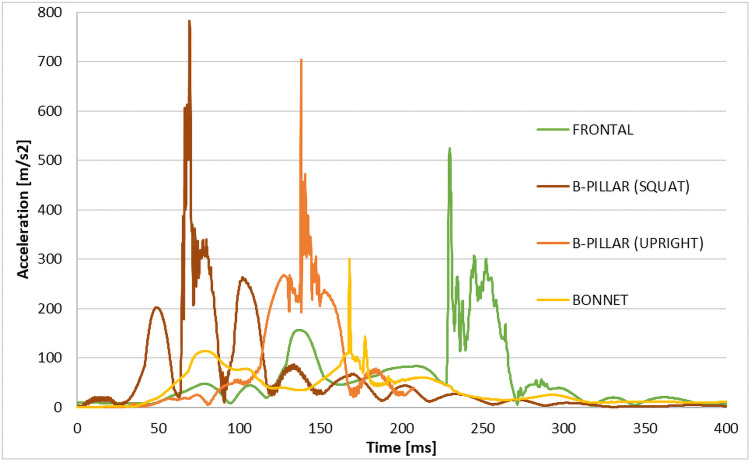
The plot of the head linear acceleration and its magnitude for the analyzed scenarios.

Based on the results of the simulations, the most dangerous accidents regarding head injuries are both of the B-pillar collisions, with the squat situation being the most dangerous among the cases studied. The main factor is that in the B-pillar crash, the head makes almost immediate contact with the car without a former arm manoeuvre, which partially absorbs the dummy’s kinematic energy. This directly contrasts the side bonnet and frontal collision, where the arm contact causes a preliminary deceleration. The arm acts as a shield for the head and thus absorbs energy that would be transformed to the head-vehicle impact. These observations correlate with the acceleration history measured in the head’s CoG and are depicted in Fig 9.

Fig 9 summarises the SES user’s head acceleration data for all of the analyzed scenarios–the CFC1000 filter was used for the plotting. The highest acceleration of 777 m/s^2^ is observed for the B-column impact scenario (squat) 69.5 ms after the impact. Successively, the maximum values of acceleration are observed for the B-pillar (upright): 703 m/s^2^ and 138 ms after the impact; frontal: 500 m/s^2^ and 230 ms after the impact, and finally bonnet: 296 m/s^2^ and 168 ms after the impact.

In each presented situation, depending on the scenario from 2 to 15 ms, after the maximal acceleration value, the second acceleration peak is observed, ranging from 42% to 66% of the value of the maximum acceleration observed for a given scenario. The authors highlight that the low rates of helmet use among electric scooter users were reported in studies, possibly leaving riders more vulnerable to head injuries [10, 37, 38].

## Conclusions

This research work aimed to investigate the kinematics of the SES user and analysis of head accelerations during an SES-vehicle crash. Electric scooters may leave users vulnerable to traumatic injuries of various severity. There is no study in the literature addressing the simulation of road accidents involving SES to the authors’ best knowledge. Currently, there are several concerns and doubts regarding the safety of the SES, along with possible regulations that can be placed to increase the safety of the scooters. This research aimed to give some insights addressing the safety of SES riders and relevant aspects for future regulation.

A total of 4 scooter-vehicle collision setups were presented throughout this research. The studied cases imply that SES user safety should become a vibrant topic in society due to the rise in the popularity of SES. Additionally, the results prove that an electric scooter crash to the automobile’s B-pillar is more dangerous than a crash over a bonnet. Although the side bonnet or frontal crash leads to a prolonged amount of time of the user being in the air, this enables the use of involuntary reflexes to protect the head. Additionally, the scooter handlebar acts as a pivot point for the rider and could be a reason for an increase in the head’s accelerations. However, despite introducing a piecewise linear plastic model of material for the scooter, the scooter behaves as a rigid body structrue with negligible plastic strains during the presented impacts. Thus, the use of a multibody model of the scooter may substitute the more complicated and time-consuming finite element model of the structure.

In future research, the kinematics of the dummy’s head could be prescribed to an actual head model to investigate the brain stress and strain or intracranial pressure. This research could strongly influence the regulations regarding the obligatory safety equipment for the SES driver and the official vehicle status.

Additionally, the reconstruction of real-world accidents involving e-scooters might be possible in the near future, thanks to accident data collection. Yang et al. [35] collected massive media reports and constructed a crash dataset including key crash elements such as rider demographics, crash type, and location. In a more recent study, Ma et al. [39] developed a mobile sensing system to collect data for quantifying the surrogate safety metrics in terms of experienced vibrations, speed changes, and proximity to surrounding objects. Compared to bicycle riding, more severe vibration events were associated with e-Scooter riding, regardless of the pavement types, which might correlate with the several cases of falls reported in the literature.

## References

[1] NACTO (2018) Shared Micromobility in the U.S.: 2018.

[2] Trivedi TK, Liu C, Antonio ALM, et al (2019) Injuries Associated With Standing Electric Scooter Use. JAMA Netw Open 2:e187381. doi: 10.1001/jamanetworkopen.2018.7381 30681711 PMC6484536

[3] Mayhew LJ, Bergin C (2019) Impact of e-scooter injuries on Emergency Department imaging. J Med Imaging Radiat Oncol 63:461–466. doi: 10.1111/1754-9485.12889 30972936

[4] Farley KX, Aizpuru M, Wilson JM, et al (2020) Estimated Incidence of Electric Scooter Injuries in the US From 2014 to 2019. JAMA Netw open 3:e2014500. doi: 10.1001/jamanetworkopen.2020.14500 32865571 PMC7489805

[5] Blomberg SNF, Rosenkrantz OCM, Lippert F, Collatz Christensen H (2019) Injury from electric scooters in Copenhagen: A retrospective cohort study. BMJ Open 9:33988. doi: 10.1136/bmjopen-2019-033988 31871261 PMC6936991

[6] Kaplan S, Vavatsoulas K, Prato CG (2014) Aggravating and mitigating factors associated with cyclist injury severity in Denmark. J Safety Res 50:75–82. doi: 10.1016/j.jsr.2014.03.012 25142363

[7] Kobayashi LM, Williams E, Brown CV., et al (2019) The e-merging e-pidemic of e-scooters. Trauma Surg Acute Care Open 4:337. doi: 10.1136/tsaco-2019-000337 31565677 PMC6744075

[8] Störmann P, Klug A, Nau C, et al (2020) Characteristics and Injury Patterns in Electric-Scooter Related Accidents—A Prospective Two-Center Report from Germany. J Clin Med 9:1569. doi: 10.3390/jcm9051569 32455862 PMC7290505

[9] Austin Public Health (2019) Dockless Electric Scooter-Related Injuries Study. Austin.

[10] Toofany M, Mohsenian S, Shum LK, et al (2021) Injury patterns and circumstances associated with electric scooter collisions: a scoping review. Inj Prev injuryprev-2020-044085. doi: 10.1136/injuryprev-2020-044085 33707220 PMC8461400

[11] OECD/ITF (2020) Safe Micromobility. Corporate Partnership Board Report.

[12] Fernandes FAO, Alves de Sousa RJ, Ptak M (2018) Application of Numerical Methods for Accident Reconstruction and Forensic Analysis. 59–98.

[13] Ptak M, Wilhelm J, Sawicki M (2019) Safety analysis of children transported on bicycle-mounted seat during traffic accidents. Int J Crashworthiness 0:1–16. doi: 10.1080/13588265.2019.1626967

[14] Xu J, Shang S, Yu G, et al (2016) Are electric self-balancing scooters safe in vehicle crash accidents? Accid Anal Prev 87:102–116. doi: 10.1016/j.aap.2015.10.022 26656151

[15] Carter EL, Neal-Sturgess CE (2009) MADYMO Reconstruction of a Real-World Collision between a Vehicle and Cyclist. Int J Crashworthiness 14:379–390. doi: 10.1080/13588260902823999

[16] Simms CK, Wood DP (2006) Effects of pre-impact pedestrian position and motion on kinematics and injuries from vehicle and ground contact. Int J Crashworthiness 11:345–355. doi: 10.1533/ijcr.2005.0109

[17] Peng Y, Chen Y, Yang J, et al (2012) A study of pedestrian and bicyclist exposure to head injury in passenger car collisions based on accident data and simulations. Saf Sci 50:1749–1759. doi: 10.1016/j.ssci.2012.03.005

[18] FKA (2019) Scooter Crash Test.

[19] Kubiczek J, Hadasik B (2020) Segmentation of the electric scooter market in Poland. Econometrics 24:50–65. doi: 10.15611/eada.2020.4.04

[20] Ptak M, Czerwińska D, Wilhelm J, et al (2019) Head-to-bonnet impact using finite element head model.

[21] National Highway Traffic Safety Administration (NHTSA) (2015) Traffic Safety Facts: 2015. US Dep Transp 1–9. DOT HS 812 409

[22] Ptak M (2019) Method to assess and enhance vulnerable road user safety during impact loading. Appl Sci 9. doi: 10.3390/app9051000

[23] Wilhelm J (2020) Injury biomechanics of bicycle-transported children during traffic accidents and the application of cork material to protect the child’s head. Wroclaw Univiersity of Science and Technology.

[24] Ptak M, Wilhelm J, Sawicki M, Rusiński E (2019) Child safety on various bicycle-mounted seats during vehicle impact. Transport 34. doi: 10.3846/transport.2019.9083

[25] Simms C, Wood D (2009) Pedestrian and Cyclist Impact. Springer Netherlands, Dordrecht.

[26] TNO (2013) MADYMO Human Body Models Manual vol 7.5.

[27] Ptak M, Wilhelm J, Klimas O, Reclik G (2019) Numerical Simulation of a Motorcycle to Road Barrier Impact. Springer Nat Switz 1–9. doi: 10.1007/978-3-030-04975-1_65

[28] Gao W, Bai Z, Li H, et al (2020) A study on cyclist head injuries based on an electric-bicycle to car accident reconstruction. Traffic Inj Prev 21:563–568. doi: 10.1080/15389588.2020.1821882 33052728

[29] Ishikawa H (1993) Impact Model for Accident Reconstruction—Normal and Tangential Restitution Coefficients.

[30] Kajzer J, Cavallero C, Bonnoit J, et al (1993) Response of the knee joint in lateral impact: Effect of bending moment. SAE Int 1993–13–00:105–116.

[31] Shang S, Masson C, Py M, et al (2020) An assessment of a multi-body pedestrian model versus PMHS during ground contact. Ircobi 89–92.

[32] Han Y, Li Q, Qian Y, et al (2018) Comparison of the landing kinematics of pedestrians and cyclists during ground impact determined from vehicle collision video records. Int J Veh Saf 10:212. doi: 10.1504/IJVS.2018.097708

[33] Zou T, Shang S, Simms C (2019) Potential benefits of controlled vehicle braking to reduce pedestrian ground contact injuries. Accid Anal Prev 129:94–107. doi: 10.1016/j.aap.2019.05.008 31132748

[34] Shang S, Masson C, Llari M, et al (2021) The predictive capacity of the MADYMO ellipsoid pedestrian model for pedestrian ground contact kinematics and injury evaluation. Accid Anal Prev 149:105803. doi: 10.1016/j.aap.2020.105803 33186825

[35] Yang H, Ma Q, Wang Z, et al (2020) Safety of micro-mobility: Analysis of E-Scooter crashes by mining news reports. Accid Anal Prev 143:105608. doi: 10.1016/j.aap.2020.105608 32480017

[36] Shah NR, Aryal S, Wen Y, Cherry CR (2021) Comparison of motor vehicle-involved e-scooter and bicycle crashes using standardized crash typology. J Safety Res 77:217–228. doi: 10.1016/j.jsr.2021.03.005 34092312

[37] Chybowski L, Przetakiewicz W (2020) Estimation of the Probability of Head Injury at a Given Abbreviated Injury Scale Level by Means of a Fuction of Head Injury Criterion. Syst Saf Hum—Tech Facil—Environ 2:91–99. doi: 10.2478/czoto-2020-0012

[38] Kaczyński P, Ptak M, Fernandes FAO, et al (2019) Development and testing of advanced cork composite sandwiches for energy-absorbing structures. Materials (Basel) 12. doi: 10.3390/ma12050697 30818808 PMC6427594

[39] Ma Q, Yang H, Mayhue A, et al (2021) E-Scooter safety: The riding risk analysis based on mobile sensing data. Accid Anal Prev 151:105954. doi: 10.1016/j.aap.2020.105954 33360874

